# Complete chloroplast genome of the *Meconopsis quintuplinervia* (Papaveraceae), a traditional medicine of Tibetan

**DOI:** 10.1080/23802359.2019.1629343

**Published:** 2019-07-12

**Authors:** Bei Xu, Hengbo Kang, Shengxiang Luo, Qian Yang, Zhe Zhao, Fangfang Peng, Zhan-Lin Liu

**Affiliations:** Key Laboratory of Resource Biology and Biotechnology in Western China (Ministry of Education), College of Life Sciences, Northwest University, Xi’an, China

**Keywords:** *Meconopsis quintuplinervia*, chloroplast genome, phylogeny

## Abstract

As a medicinal herb of Tibetan, *Meconopsis quintuplinervia* is often utilized for treating pneumonia. In this study, the complete chloroplast genome of *M. quintuplinervia* was determined by next-generation sequencing technology. The overall genome was 154,997 bp in size, including a large single copy (LSC), a small single copy (SSC) and two inverted repeat (IR) regions, which were 85,153 bp, 17,876 bp, and 25,984 bp in length, respectively. The circular chloroplast genome owned 129 genes, comprising 84 protein-coding genes, 8 ribosomal RNA genes (four rRNA species), and 37 transfer RNA genes. The GC contents of the entire sequence, LSC, SSC, and IR region were 38.5%, 37.1%, 32.8%, and 43%, separately. The maximum likelihood tree revealed that *M. quintuplinervia* was closely related to *M. racemosa* with strong support value.

*Meconopsis* Vig., mainly distributed in Himalaya and southwest China, consists of about 50–60 alpine species and harbors high morphological diversity providing widely desirable resources for horticulture (Xiao and Simpson 2015). But, for the lack of effective markers, the phylogenetic position of the genus and interspecific relationships have been contentious. Owing to the conservation and maternal inheritance (Ravi et al. 2008), chloroplast genome has been a useful tool for exploring species evolution and phylogenetic relationships. In this study, we reported the complete chloroplast of *M. quintuplinervia*, commonly used as a Tibetan medicine to treat pneumonia in the Qinghai-Tibet plateau, which would provide new genetic resources for the research of *Meconopsis*.

Samples of *M. quintuplinervia* were collected from Taibai Mountain (107.77°E, 33.95°N, 3200m), China, and the voucher (Liu2016TB017) was deposited at the Evolutionary Botany Laboratory (EBL), Northwest University. Genomic DNAs were isolated from the fresh leaves and sequenced in the Illumina HiSeq 2500 platform. Raw reads were trimmed by NGSQC Toolkit v2.3.3 (Patel and Jain 2012) and assembled using MIRA 4.0.2 (Chevreux et al. 2004). The cp genome was annotated by Geneious R8 (Biomatters Ltd., Auckland, New Zealand) with modification by using DOGMA (Wyman et al. 2004). The maximum likelihood (ML) tree was constructed with six species in Papaveraceae, two species in Circaeasteraceae, Lardizabalaceae, and Brassicaceae, respectively.

The cp genome sequence of *M. quintuplinervia* (GenBank accession number MK801686) is 154,997 bp in length and has a quadripartite structure including a large single copy (LSC) region of 85,153 bp, a small copy (SSC) region of 17,876 bp and two inverted repeat (IR) copies of 25,984 bp. The total GC content is 38.5%, and the corresponding values of LSC, SSC, and IR are 37.1%, 32.8%, and 43%, respectively. There are 129 genes in the circular cpDNA, containing 84 protein-coding genes, eight ribosomal RNA genes (four rRNA species), and 37 transfer RNA genes. The gene of *trn*S occurs in three copies and two genes (*trn*G & *trn*T) are duplicated in the LSC region. The ML tree indicated that *M. quintuplinervia* was closely related to *M. racemosa* and the genus of *Meconopsis* was sister to *Papaver* with a high support value (Figure 1). This chloroplast genome information offers available markers for the phylogenomics and population genetics works in *Meconopsis* and the family of Papaveraceae.

**Figure 1.**
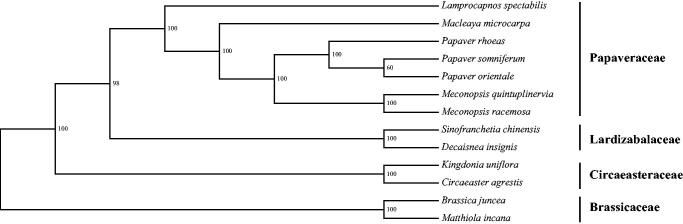
Maximum likelihood phylogenetic tree based on complete chloroplast genome sequences from twelve species. Accession numbers: *Lamprocapnos spectabilis* NC_039756, *Macleaya microcarpa* NC_039623, *Papaver rhoeas* NC_037831, *Papaver somniferum* NC_029434, *Papaver orientale* NC_037832, *Meconopsis racemosa* NC_039625, *Sinofranchetia chinensis* NC_041488, *Decaisnea insignis* NC_035941, *Kingdonia uniflora* NC_035873, *Circaeaster agrestis* NC_035872, *Brassica juncea* NC_028272 and *Matthiola incana* KY912030.
